# Contained or represented? The varied consequences of reserved seats for emigrants in the legislatures of Ecuador and Colombia

**DOI:** 10.1186/s40878-018-0101-7

**Published:** 2018-12-20

**Authors:** Pau Palop-García

**Affiliations:** GIGA German Institute of Global and Area Studies, Hamburg, Germany

**Keywords:** Reserved seats, Descriptive representation, Non-resident citizens, Emigrants

## Abstract

The legislatures of Colombia and Ecuador have reserved seats for their non-resident citizens (emigrants). This paper analyses the relationship between the formal, descriptive, and substantive dimensions of emigrant representation in their homeland legislatures. The analysis compares the legislative work of emigrant MPs (EMPs) with the legislative work of non-emigrant MPs (NEMPs) in Ecuador and Colombia. It presents a mixed methods approach that combines a quantitative text analysis based on an original dataset –composed of 35,446 floor speeches– with in-depth interviews with six EMPs. The results show that emigrant-related issues are significantly more salient in the legislature of Ecuador and Colombia suggesting that the effect of emigrant-reserved seats is correlated to the size of the external district. Furthermore, the analysis reveals that EMPs have a ‘mixed agenda’ composed by emigrant and domestic-related issues. Finally, the article shows that the probability of classifying a speech as emigrant-related increases when it is given by an EMP and not a NEMP. This effect is stronger in Ecuador than in Colombia. All in all, the article shows evidence that configurations that allocate several EMPs are more efficient in achieving substantive representation.

## Introduction

In 1991, Colombia included a provision in its new Constitution that granted non-resident citizens the possibility to elect their own representatives in the lower legislative house. In 2008, almost two decades after Colombia’s constitutional change, Ecuador also extended active and passive electoral rights to non-residents, reserving six special seats for emigrants MPs (hereafter, EMPs) in the National Assembly. Up to 2018, 15 countries in the world have created special seats for emigrants in their legislative houses (Collyer, 2014; “Les Sénégalais de l'étranger seront,” 2017; Présidence de la République du Niger, 2018) and even more states are discussing adopting such a mechanism (e.g. Peru, Spain, Uruguay or Jamaica). The inclusion of non-resident citizens into the homeland legislative houses constitutes a major innovation in terms of how states conceive the boundaries of their polity and how they shape their relationship with their citizens abroad. However, there is scarce information about how these mechanisms of representation of emigrants work. Does the special representation of emigrants guarantee the inclusion of non-resident citizens into their homeland political legislatures? Or, on the contrary, are emigrant special seats a mechanism designed to contain the political influence of non-resident citizen populations?

In this paper I contribute to answering these questions by comparing the role that Ecuadorian and Colombian EMPs undertake at the legislative level. The general research question of this paper examines whether or not the presence of emigrants in parliaments translates into a responsive outcome to the concerns, interests and stakes of non-resident citizens in the work of the legislative chambers. To use the terminology proposed by Pitkin (1967), this paper analyzes whether or not the descriptive representation of emigrants in the legislative chambers translates into substantive representation of an emigrant agenda. Representing the emigrant agenda is not, however, a straightforward task since the interests of diasporas may vary, not only among different countries of origin, but also among countries of destination. Nevertheless, delimiting the contours of the emigrant agenda is a necessary step to answer this research question (I tackle this issue in detail in the methodological section).

The theoretical starting point of this paper is located at the intersection of two strands of literature that so far have not been sufficiently linked. On the one hand, there is a vast literature that studies the effect of quotas and the reservation of seats on the inclusion of underrepresented groups in the legislative process (Htun, 2016; Reynolds, 2005). For example, scholars have studied the representation of indigenous populations (Crisp, Demirkaya, & Millian, 2014), women (Lovenduski & Norris, 2003; Mansbridge, 1999; Schwindt-Bayer, 2010), ethnic minorities (Hänni, 2016; Htun, 2016; Minta, 2009; Rouse, 2013) and immigrants in states of reception (Bird, Saalfeld, & Wust, 2010; Saalfeld, 2011). To my knowledge, however, scholars working on political representation have not applied its methodologies nor theoretical framework to the analysis of emigrant political representation. This paper intends to fill this gap in the literature. On the other hand, migration scholars concerned with external voting have investigated the specificities and commonalities of external electoral systems (Escobar, 2007), emigrant voting behavior (Burgess, 2014; Lafleur & Sánchez-Domínguez, 2014) and the normative implications of external voting rights (Bauböck, 2009; Rubio-Marin, 2006). However, it appears that the literature of external voting has suffered from a bias towards the analysis of active external rights, leaving, with some exceptions (Collard, 2013; Collyer, 2014; Østergaard-Nielsen & Ciornei, 2017), emigrant passive electoral rights under-researched.

This study is based on a mixed methods approach that combines an original dataset of 35,446 parliamentary speeches given by EMPs and Non-Emigrant MPs (hereafter, NEMPs), with in-depth interviews of six EMPs. In the analysis, I test whether the presence of emigrant special representatives in the legislative houses has an impact on the substantive representation of emigrant-related issues. In addition to this, I test whether the different configurations of emigrant special representation that exist in Ecuador and Colombia are significant in explaining the substantive representation of emigrant-related issues.

## Theoretical framework and hypotheses

The theoretical background of this paper is built upon two different strands of the literature that, until now, have not been sufficiently interconnected: the literature on political representation and the literature on political transnationalism.

### The descriptive-substantive link of emigrant representation

This paper takes the multidimensional concept of ‘political representation’ as proposed by Pitkin (1967) as a theoretical starting point. Some scholars have argued that the dimensions of representation should not be studied in isolation, but in a cohesive manner through an integrated model (Schwindt-Bayer & Mishler, 2005). Following this advice, this paper features an analysis of the link between the formal, descriptive, and substantive representation of emigrants in their states of origin. That is to say, the relation between the rules used to appoint the representatives, the characteristics of the representatives and the responsiveness of the work conducted by the representatives.

Classic studies in political sciences have studied, for instance, the effect of district size on representation or the differences between majoritarian and proportional electoral designs (Rae, 1967). There has also been extensive research on the effects of other aspects of electoral systems, such as quotas or reserved seats, on the descriptive representation of minority groups and the substantive representation of their interests and stakes (Collard, 2013; Htun, 2016; Krook & Norris, 2014). The core question addressed by this literature refers to the influence that the presence of a minority in a legislature holds over substantive representation. The answer to this question is not straightforward and is affected by several variables. For example, in a recent comparative study on the representation of 88 minority groups of 47 countries, Hänni concludes that descriptive representation has a stronger effect on policy outcomes if the minority representatives are also included in government, the legislature is strong, and are backed up by a numerous group (Hänni, 2016). Zuber (2015) demonstrated that political parties may also play a significant role in shaping the outcome of descriptive representation. Minta, in his study about the relationship between legislative oversight and the representation of black and Latino interests in the US Congress, observed that descriptive representation had a significant effect on the racial policy dimension, but not in other policy dimensions, such as welfare (Minta, 2009, p. 210). Saalfeld, in his study of the parliamentary questions asked by ethnic-minority legislators in the British House of Commons, proved that MPs with a minority background do ask more questions related to migrant and ethnic issues than the rest of the MPs. However, his results also showed that all MPs in his sample, regardless of their background, were responsive to the demographic characteristics of their districts (Saalfeld, 2011). Similarly, Wüst observed in his study about the German national and state parliaments that the presence of visible minority MPs is correlated with a higher share of legislative questions related to migrant issues (Wüst, 2014). Taking into account the evidence about the descriptive-substantive link found by other scholars, I expect that *the presence of emigrant parliamentarians (EMPs) in a legislative house translates into substantive representation of emigrant-related issues in the legislative process (H1)*.

### The formal-descriptive-substantive link of emigrant representation

Studies focused on emigrant special representation are scarce within the literature of political transnationalism. In general, scholars have treated this topic as an appendix to research about external active voting. However, it is possible to find some exceptions to this trend. Lafleur (2013) inquired into the mechanisms of special representation implemented in Italy. As he stated, there are reasonable doubts about the capacity of external parliamentarians to substantively represent the heterogeneity of emigrant interests (p. 137). He argued that two things can prevent substantive representation of emigrants: party and ideological alliances (when party interests are prioritized over the interests of the emigrant constituency), and the competition between representatives with the same external constituency (pp. 137–138). In a recent study, Østergaard-Nielsen and Ciornei (2017) explained why the political parties of four European democracies pay attention to emigrants. In their analysis, the special representation of emigrants was an explanatory variable. They concluded that the introduction of emigrant special representation had a significant impact on emigration salience in party agendas (p. 23). Interestingly, they also discovered that the closeness of party competition increased the effect of special representation. However, they recognized that a study focused on parties and not individuals cannot fully explain the differences across emigration salience in different countries and at different points in time. As they put it “the extent to which and why emigrant representatives ‘take over’ the issue of emigration is worthy of further investigation” (p. 8).

Conveniently enough for the scope of this paper, the use of special seats is not limited to emigrants. In fact, this mechanism is broadly used around the world to assure the presence in legislative chambers of ethnic, racial or religious minorities (Reynolds, 2005). Research on reserved seats and quotas has shown that, not only is the presence of an underrepresented group in a given legislative house important, but so are the configurations of the representation as defined by formal rules (Reynolds, 2005). In this sense, we know that the special representation of emigrants can be configured in diverse forms, depending on the number of the seats that are reserved for emigrants or the size of the district used to elect emigrants’ special representatives (Hutcheson & Arrighi, 2015). Collyer (2014) hypothesized about the effect of the external district size for the substantive representation of emigrants. He took a stock of 13 countries with reserved seats for emigrants in at least one of their legislative houses and compared their external electoral rules. In his conclusions, he made an important point about the varied consequences of emigrant special representation. He argued that the reservation of seats for emigrants had not always been adopted to ensure their representation, but rather that it was sometimes introduced to contain the influence that a large community of emigrants could have over homeland politics. This ‘containment effect’ would be reached by reserving a number of seats to emigrants that underrepresents their total share of a given country’s population. To understand better the mechanism behind this theoretical ‘containment’ effect of emigrant special representation, we can draw upon on critical mass theory. Scholars working on the representation of minorities in legislative chambers have used this approach in the past to explain the relationship between the number of minority representatives and substantive representation. Critical mass theory commonly refers to the necessity of reaching a minimum number of individuals (critical mass) in order to produce social change (Kanter, 1993). Applied to legislative studies, it has been used to argue that a minimum number of minority representatives is indeed needed in order to accomplish substantive representation (Dahlerup, 2006). Particularly, in the case of gender studies, some scholars argued that a threshold of around 15% of the seats in a legislature is needed for women to make a difference by setting the legislative agenda or passing women-related regulation (Childs & Krook, 2008). For instance, Schwindt-Bayer (2010), in her analysis of representation of women in Latin American legislatures, concluded that in male-dominated legislatures, women were marginalized and not able to represent non- women specific issues to the same extent as men (p. 188). Following critical mass theory, I expect *that in order to achieve substantive representation and avoid a ‘containment effect’, it is necessary to reach a critical mass of emigrant parliamentarians (H2a)*.

The claims made from critical mass theory, however, have been contested by some scholars who have argued that, even in parliaments in which a given minority has not reached a ‘critical mass’, substantive representation could be still achieved. In fact, some have observed that substantive representation is greater when the minority is still significantly underrepresented in a legislature. For instance, Rouse (2013) found out that Latinos in the US state legislatures were more prompt to engage in substantive representation of a Latino agenda when their presence was an exception and not the rule (p. 67). In the same line, Bratton (2005) in her analysis of the link between descriptive and substantive representation of women in US state legislatures observed that agenda-setting discrepancies between men and women decreased in gender-balanced legislatures. In this paper, I test whether critical mass theory is able to explain the emigrant descriptive- substantive representation link or, on the contrary, that there is evidence demonstrating that the number of EMPs is not relevant. Thus, I also test whether or *not the presence of a single EMP leads to a more effective substantive representation of an emigrant agenda (H2b)*.

## Case selection

To conduct this analysis, I selected two countries, Ecuador and Colombia, that have allocated seats for emigrants in at least one of their legislative houses, but that differ in the number of seats reserved. Ecuador has six seats reserved to emigrants and Colombia between one and two.^1^ Both countries, however, share important commonalities. Beyond the most obvious, geographic location, language and similar colonial past, both have a significant part of their citizens abroad (see Table 1). It is estimated that around 7–10% of Ecuadorian and Colombians live abroad. Most Ecuadorian emigrants –around 82%– are concentrated in the US, Spain and Italy (Herrera Mosquera, Moncayo, & Escobar, 2012). Similarly, the majority of Colombians living abroad –about 75%– reside in only three countries (Venezuela, US and Spain) (Ramírez, Zuluaga, & Perilla, 2013). Both countries are also considered to be among the most liberal of the region in terms of external electoral rights and both have extended extraterritorial citizenship to the second generation (Escobar, 2007). Moreover, external voting is voluntary in the two countries (although in Ecuador is compulsory for resident voters).

**Table 1:** Key Variables for case selection

Country	Population abroad	Date of adoption	Number of EMPs	EMP/NEMP
Ecuador	3 Millions	2008	6	4.8%
Colombia	3.5 Millions	1991	1–2	0.6–1.2%

### Ecuador: a case of (almost) emigrant proportional representation

Since the *Revolución Ciudadana*, which was initiated in 2006 and consolidated with a new constitution in 2008, migration has been a highly salient topic in the political and public agenda in Ecuador. During the *Correísmo*, Ecuadorian migrants were portrayed as victims of the severe economic and political crisis that took place at the end of the 90s (Boccagni & Ramírez, 2013). Moreover, the former Ecuadorian President, Rafael Correa, has referred publicly in several occasions to the emigrant community as the ‘Fifth Region’, an entity seen as equivalent to the four territorial regions of Ecuador (Boccagni & Ramírez, 2013, S. 725). Ecuador is also a case in point of state-led transnationalism in which the expansion of the political participation mechanisms for emigrants has been led by the state of origin (Boccagni & Ramírez, 2013; Margheritis, 2016). Nevertheless, the participation of Ecuadorians abroad has grown in absolute terms with every election since they were able to participate, the first time being in 2006. Numbers increased from around 143,000 registered voters to 380,512 registered voters (34.96 turnout) in the first round of the 2017 elections (Consejo Nacional Electoral, 2018).

The Constitution created a unicameral legislature, with a National Assembly composed of 124 representatives^2^ elected in regional districts (103), in a single-national district (15) and in three districts allocated outside the territorial boundaries of Ecuador (6, Europe and Oceania, United States and Canada, and Latin America and the Caribbean). In total, the number of seats reserved for emigrants accounted for 4.8% of the total seats of the Ecuadorian assembly^2^ (see Table 1). Thus, the share of the population represented by an emigrant seat in the National Assembly is indeed similar to the number represented by a resident seat.

The analysis includes a total of 10 Ecuadorian EMPs (2 of them were reelected for the second legislative period included in the analysis). Thus, it includes a mix of MPs with more and less seniority (the maximum number of periods that Ecuadorian MPs can serve is two). Also, all of them were members of Alianza PAIS, the political movement in the Ecuadorian government. To stand as a candidate for an emigrant seat, the Ecuadorian law requires previous residence abroad. Thus, all EMPs are members of the Ecuadorian diaspora. Nevertheless, during the time they serve in the National Assembly, they move back to Quito, as they are required to be present in the legislative debates.

### Colombia: a case of emigrant underrepresentation

Colombia started to adopt emigrant policies much earlier than Ecuador. In fact, Colombia was one of the first countries in the region that granted political rights to non-residents when in 1962 emigrants were allowed to vote for presidential elections (Bermúdez, 2014). Thirty years later, with the Constitution of 1991, the country also granted emigrants passive and active electoral rights in legislative elections. However, the implementation of passive electoral rights was delayed until 2002, when Colombians living abroad were able to run for the first time for a reserved seat in the Colombian House of Representatives (González, 2010). In Colombia, unlike the situation in Ecuador, emigrants are significantly underrepresented in the lower house. The Colombian House of Representatives only has one or two reserved seats for emigrants^3^ who are elected in a single external district (representing between 0.6 and 1.2% of the total seats^4^). Thus, in Colombia, the size of the population represented by EMPs is significantly higher that the size of the population represented by Non-Emigrant Members of the Parliament (NEMPs). Additionally, Colombian emigrant representatives are part of the five seats reserved to minorities by the constitution and distributed between representatives of the ethnic communities, political minorities and emigrants. Contrary to Ecuador, it is clear in the case of Colombia that the legislator did not conceive emigrant special representation as the territorial representation of a *sui generis* external territorial district, but as the inclusion of a minority that otherwise would be excluded from the Colombian House of Representatives.

Furthermore, the migration profile of Colombia is different from Ecuador’s. Through three migration waves dating back to the mid- 60s, Colombians have emigrated for different reasons, including economic crises and the instability caused by the armed conflict and related violence (Maisonave & Ortí, 2010). Participation in elections from abroad in Colombia is significantly lower than in Ecuador. In the last legislative elections held in 2014, the number of external voters was approximately of 48,000 (out of a total census of 571,420).^5^

The Colombian representatives included in the analysis are somewhat more diverse than in the Ecuadorian case. One of them was reelected and is part of the coalition that was in power in Colombia during the two legislative periods analyzed. This EMP also had previously served as a consul in New York. Another second EMP was a member of MIRA, one of the opposition parties. Unlike the other EMP, she used to reside in Spain and had no ties with any Colombian institutions before her election. Colombian EMPs are required to be present in Bogota for the floor sessions.

## Measurements and methods

### Dependent variable

In this research, the dependent variable is the substantive representation of emigrants. I argue that non-resident citizens are substantively represented in the parliaments of Ecuador and Colombia as long as their agenda is represented. Drawing upon the study of Østergaard-Nielsen and Ciornei (2017), when deciding which issues to include in the ‘emigrant agenda’, I include (1) the status of non-resident citizens, for instance, the regulation of dual nationality; (2) policies that aim at assisting non-residents abroad, such as the improvement in consular services; (3) policies that foster the integration of emigrants in the state of origin, for example, the creation of a consultative body to discuss emigrant issues, and (4) policies oriented to foster the return of migrants.

Although the basic definition of substantive representation provided by Pitkin in her seminal work, “acting in the interest of the represented in a manner responsive to them” (Pitkin, 1967, p. 209), leaves us with plenty of room for interpretation, it is generally accepted that substantive representation goes beyond mere policy responsiveness (i.e. the enactment of laws) (Schwindt-Bayer, 2010). In fact, scholars working on the representation of minorities have measured the work conducted by minority representatives in many different ways. Eulau and Karps (1977), for instance, argued that the concept also entailed service, allocation and symbolic responsiveness. Fenno (1977) shifted the focus from the work conducted in the legislatures to the work carried out in the districts of origin, what he defined as ‘home style’ representation. In this paper, I use two indicators of substantive emigrant representation. Firstly, I consider the number of floor speeches given by EMPs and NEMPs. This variable allows me to analyze if EMPs are marginalized within the legislatures in comparison to NEMPs. Secondly, I use the number of floor speeches related explicitly to the emigrant agenda. I analyze floor sessions for two reasons. Firstly, it could be said that they represent the moment of the legislative process with more visibility. Secondly, they are a good indicator of the priorities of the representatives’ agendas, since opportunities for a representative to participate in floor sessions are scarce.^6^

It is not the aim of this article to compare the representation of non-resident citizens between legislative periods with and without emigrant special seats, but to assess the work of EMPs in comparison to NEMPs and to analyze to what extent the representation of an emigrant agenda in a legislature is dependent on the work conducted by EMPs. For this reason, I study two completed legislative periods with emigrant representation for each country (2010–2014 and 2014–2018 for Colombia; and 2009–2013 and 2013–2017 for Ecuador). To measure the second indicator of emigrant substantive representation, I apply quantitative text analysis based on dictionary coding, which is a supervised approach successfully implemented by other scholars to code a large sets of documents with high reliability (Langer & Sagarzazu, 2015). One of the most extended applications of dictionary coding is the identification of issue areas based on a set of tokens (words) defined beforehand (Pardos-Prado & Sagarzazu, 2016). Moreover, previous research based on automatic text classification have proven valid to study substantive representation of underrepresented groups (see for instance Saalfeld, 2011). The most crucial step in this methodology is the definition of the tokens included in the dictionary. To achieve this goal, I firstly interviewed four EMPs from Ecuador and two from Colombia and asked them which issues they considered to be relevant in their legislative work as emigrant representatives; and secondly, I analyzed the content of a sample of speeches given by EMPs that were identified as related to emigrant issues in a hand-coded sample of speeches (see Table 2). Once I had the dictionary for each country, I ran a recognition program that allowed me to classify the speeches included in the dataset automatically. A speech is coded as 0, if it is not explicitly related to an emigrant issue and 1 if it is. Finally, I inspected the validity of the automated classification by comparing the results of hand-coding and dictionary coding of a randomly selected sample of speeches given in each legislature (see Appendix for more information about the methodology).^7^

**Table 2:** Dictionary used to code speeches. Tokens translate by author from the original versions (in Spanish). Detailed list of tokens used available on demand. Source: Author’s own

Ecuador	Colombia
“Ecuadorians living abroad”, “persons in situation of human mobility”, “remittances”, “emigration”/“emigration”, “migrant”/“migration”, “returnees”, “universal citizenship”/“dual nationality”, “consular network”/“consular services”	“Colombians living abroad”, “victims abroad”/“refugees abroad”, “national migration system”, “emigration”/“emigration”, “migrant”/“migration”, “Law of return”/“1465”, “double nationality”, “consular network”/“consular services”

The analysis includes a total of 35,446 speeches and covers 355 floor sessions of Ecuador for the period 2009–2013 and 307^8^ sessions for the period 2013–2017; and 295 floor sessions of Colombia for the period 2010–2014 and 272 for the period 2014–2018 (see descriptive statistics in Table 3).

**Table 3:** Descriptive statistics. Source: Author’ own

	Ecuador				Colombia			
	Period 2009–2013		Period 2013–2017		Period 2010–2014		Period 2014–2018	
	N	Percentage	N	Percentage	N	Percentage	N	Percentage
Emigrant speech								
Yes	124	1.5	197	4.7	34	0.3	96	0.9
No	8,479	98.6	4,018	95.3	11,646	99.7	10,851	99.1
EMP								
Yes	312	3.6	148	3.5	128	1.1	187	1.7
No	8,292	96.4	4,067	96.5	11,552	98.9	10,760	98.3
EDMP								
EDMP	1,664	19.3	1003	23.8	3,543	30.3	3511	32.1
EMP	312	3.6	148	3.5	128	1.1	187	1.7
Regular MP	6,628	77.1	3,064	72.7	8,009	68.6	7,249	66.2
Sex								
Women	2,013	23.4	1,580	37.5	1,851	15.9	1,555	14.2
Men	6,591	76.6	2,635	62.5	9,829	84.1	9,392	85.8
Party in government								
Yes	3,652	42.5	2,531	60.0	7,996	68.5	4,465	40.8
No	4,952	57.6	1,684	40.0	3,684	31.5	6,482	59.2
Rol								
Yes	1,317	15.3	709	16.8	11,227	96.1	827	7.6
No	7,287	84.7	3,506	83.2	453	3.9	10,120	92.4
*Total*	8,604	100	4,215	100	11,680	100	10,947	100

### Explanatory variable

The main explanatory variable in this study is the membership of individual MPs to the group of EMPs or to the group of NEMPs. I use a dichotomous variable, coded 0 for NEMPs and 1 for EMPs. In addition to this, I also test whether the membership of MPs to districts highly affected by emigration (hereafter, EDMP) affects the substantive representation of emigration-related issues in the speeches of the individual MPs in the floor discussion. I assume that MPs representing these districts may also have an interest in representing the emigrant community, since a significant part of the well-being of their constituency is shaped by the access to remittances. To this end, I use a nominal variable composed of three categories: NEMP (coded as 0), EMP (coded as 1) and EDMP (coded as 2). To define the districts that are highly affected by emigration, I use the distribution of remittances across regions. Districts receiving more than 5% of the total country remittances are coded as highly affected by emigration.^9^

### Control variables

The substantive representation of emigrant-related issues in floor speeches could be also affected by other variables that, for this particular research, are included as controls. Firstly, it is also possible that the performance of individual MPs is conditioned by their relationship with the party in government. EMPs representing a party that is also part of the government will have less interest of mentioning emigrant issues. MPs are code as 0 if they represent a party in the opposition and 1 if they represent a party in the government.^10^ Secondly, it is also plausible that the number and the content of interventions are affected by the role that MPs assume in the legislatures. Thirdly, previous research has proved that women could be marginalized within the legislative houses in comparison to men and therefore intervene less in floor debates (Schwindt-Bayer, 2010). To bear this effect in mind, I also include the gender of MPs as a control. Finally, I control by the legislative period since it is possible that the content of the interventions could be affected by the general political and social context of the legislative period.

### Methods

In this analysis, I implement a mixed methods approach (Johnson, Onwuegbuzie, & Turner, 2007). In the first part of the study, I test quantitatively test (using descriptive statistics and logistic regression models) whether there are significant differences between the number of interventions of EMPs and NEMPs and between the number of interventions dedicated to emigrant issues of EMPs and NEMPs. In the second step of the analysis, I validate the findings of the quantitative analysis with the qualitative information provided by the interviews that I conducted with EMPs of Colombia and Ecuador. The interviews were conducted between April and May 2016 in Quito and Bogota and were based on the same questionnaire (see Table 4).

**Table 4:** Methodological steps and data sources. Source: Author’s own

Methodology				
Data sources	Creation of dictionary	Dictionary coding	Quantitative analysis	Qualitative analysis
Interviews with EMPs	Identification emigrant issues			Validation and identification of mechanisms
Floor speeches	Identification emigrant words	Codification of speeches	Cross-tabulation and logistic regression	

## Results

### Number of interventions per MP

**Fig. 1:**
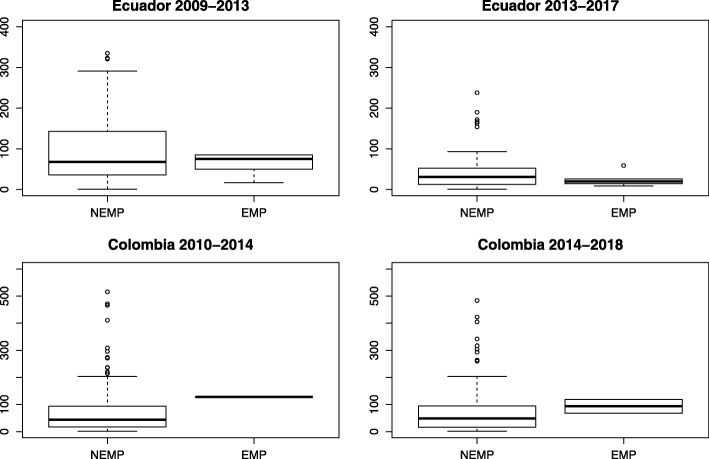
Interventions of EMPs and NEMPs in floor discussions. Source: Author’s own

The first indicator that gives us information about the substantive representation of emigrants, in regard the parliamentary work conducted by their special representatives, is the total number of floor interventions of EMPs. Figure 1 shows that the Colombian EMPs participated significantly more than the average MPs in Colombia. In the period 2010–2014, the EMP gave a total of 128 speeches whereas the NEMPs participated in average 81.93 times during the floor discussions (sd = 105.95). In the period 2014–2018, the EMPs participated in the floor sessions on average 93.5 times (sd = 36.06), while the NEMPs participated in average 74.72 times (sd = 87.59). In the case of Ecuador, as shown in Fig. 1, there is significant variation in the number of interventions of EMPs. In both legislative periods analyzed, their average participation is lower (in 2009–2013, mean = 62.4; sd = 29.14; and in 2013–2017, mean = 24.67, sd = 17.97) than the average participation of NEMPs (in 2009–2013, mean = 97.55; sd = 86.12; and in 2013–2017, mean = 42.81, sd = 44.68). Nevertheless, the fact that there is significant variance within the EMP group in both periods suggests that there are other variables that explain the number of interventions given by Ecuadorian MPs, such as the position within the party hierarchy or seniority. All in all, since there is not a clear pattern, the findings do not confirm or refute the hypotheses H2a and H2b.

### Number of emigrant-related interventions per floor session

**Fig. 2:**
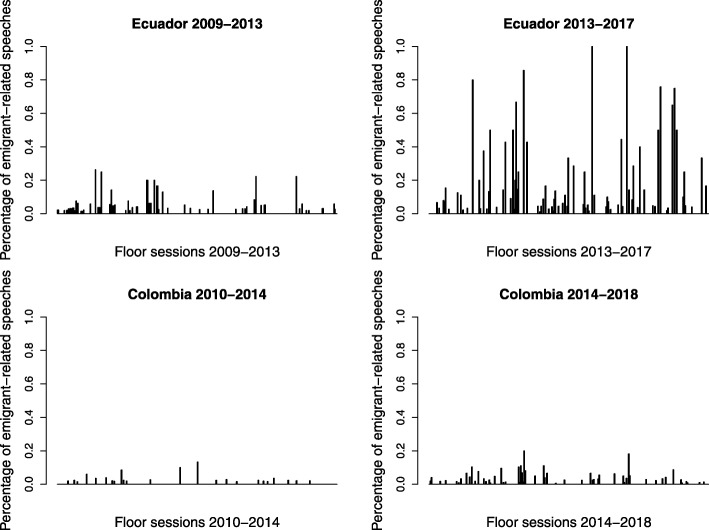
Percentage of speeches related to emigration in plenary sessions. Author’s own

Figure 2 shows the salience of emigrant issues in the plenary sessions of Colombia (2010–2014 and 2014–2018) and Ecuador (2009–2013 and 2013–2017). Each bar represents the relative number of speeches that were recognized as being connected to the emigrant agenda, within the total number of speeches given in a floor session (scores, therefore, range from 0 to 1, being 0 that no emigrant-related speech has been given and 1 that all the speeches given in the floor session were related to emigration). The graph shows interesting findings. First, the salience of emigrant issues is higher in Ecuador than in Colombia in all the periods analyzed. Second, for both Ecuador and Colombia, in the second legislative period of analysis the salience of emigrant-related issues is higher. Furthermore, the graph shows that, during the period 2013–2017, there are floor sessions in Ecuador that are fully dedicated to emigrant-related issues. This probably reflects the importance of the Human Mobility Law that was introduced by the Ecuadorian EMPs during the period 2013–2017 and became an important issue of the legislative agenda. All in all, the findings draw from this analysis are consistent with the expectation that a greater number of EMPs increases the overall salience of emigrant issues in floor discussions (H2a).

### Determinants of emigrant-related speeches

The second indicator that I use to analyze the work conducted by EMPs is the number of interventions dedicated to the emigrant agenda. Firstly, I conduct a cross-tabulation (Table 5) with the dependent variable (a dichotomous indicator that classifies the floor interventions as explicitly related to an emigrant agenda or not) and the main explanatory variable (membership to the group of emigrant special representatives). The results of the cross-tabulation lead to some important conclusions. In Colombia, NEMPs is the group that, in absolute and relative terms, talked most about emigrant issues: during the period 2010–2014, 76.5% of the emigrant-related speeches in Colombia were given by NEMPs, while only 23.5% were given by the EMP; and during the period 2014–2018, 67.7% of the emigrant-related speeches were given by NEMPs and 32.3% by EMPs. In Ecuador, interestingly, there is not a clear pattern. During the period 2009–2013, 60.0% of the emigrant-related speeches were given by EMPs, while only 40.0% by NEMPs. However, during the period 2013–2017, 65.5% of the emigrant-related speeches were given by NEMPs and only 34.5% by EMPs.

**Table 5:** Speeches explicitly related to emigrant issues classified by migrant background (Ecuador and Colombia). Total number and column percent. Source: Author’ own

Country	Period	MP	NO	YES	TOTAL
Ecuador	2009–2013	NEMP	8,242	50	8,292
			(97.2%)	(40.0%)	(96.4%)
		EMP	237	75	312
			(2.8%)	(60.0%)	(3.6%)
		Total	8,479	125	8,604
		*Chi-square = 1153.5. p-value < 0.01			
Ecuador	2013–2017	NEMP	3.938	129	4,067
			(98.0%)	(65.5%)	(96.5%)
		EMP	80	68	148
			(2.0%)	(34.5%)	(3.5%)
		Total	4,018	197	4,215
		*Chi-square = 586.4. p-value < 0.01			
Colombia	2010–2014	NEMP	11,526	26	11,552
			(99.0%)	(76.5%)	(98.9%)
		EMP	120	8	128
			(1.0%)	(23.5%)	(1.1%)
		Total	11,646	34	11,680
		*Chi-square = 158.33. p-value < 0.01			
Colombia	2014–2018	NEMP	10,695	65	10,760
			(98.6%)	(67.7%)	(98.3%)
		EMP	156	31	187
			(1.4%)	(32.3%)	(1.7%)
		Total	10,851	96	10,947
		*Chi-square = 539.5. p-value < 0.01			

This raises an important question: are EMPs more focused on representing the interests of their constituents living abroad than NEMPs? As Tables 5 shows, this seems to be the case. In total, during the period 2009–2013, 75 out of the 312 speeches given by Ecuadorian EMPs were explicitly related to an emigrant agenda and only 50 speeches out of 8,242 given by NEMPs addressed matters related to emigration. This pattern is even clearer during the period 2013–2017, when 68 speeches out of the 148 given by EMPs and 129 out of 3,938 given by NEMPs were dedicated to emigrant issues. In Colombia EMPs also focus relatively more on emigrant issues than NEMPs. During the period 2010–2014, 8 out of 128 speeches given by EMP focused on emigrant issues (and only 26 out of 11,552 of the speeches given by NEMPs). In the same fashion, during the period 2014–2018, 31 out of 187 speeches given by EMPs were focused on emigrants, while only 65 out of 10,760 of the speeches given by NEMP addressed emigrant issues.

In order to explore this specific issue further, I calculate four models using binary logistical regressions (see Table 6). The main aim of these models is to assess what is the impact of being an EMP or a NEMP on the content of the speech given. In other words, if being an EMP increases the odds of giving an emigrant-related speech. I calculate two models for each country.^11^ The first set includes the explanatory variable as dichotomous (EMP/NEMP) and the second, the explanatory variable as a nominal indicator with three categories (EMP, EDMP and NEMP). Since there is a class bias (the proportion of emigrant-related speeches is much smaller than the proportion of non-emigrant speeches), I randomly subset the samples to create a development sample and a validation sample. The development data used to calculate the models is composed by 50% of emigrant- related speeches and 50% of non-emigrant-related speeches. The explanatory power of the models based on the development data is then validated with the rest of the data.

**Table 6:** Logistic regression for the classification of speeches: Non-emigrant related speeches (0). emigrant-related speeches (1)

	Ecuador		Colombia	
	Model 1a	Model 1b	Model 2a	Model 2a
EMP	4.300^***^	4.325^***^	2.696^***^	2.714^***^
	(0.625)	(0.627)	(0.683)	(0.688)
EDMP		0.134		0.087
		(0.301)		(0.382)
Period	1.613^***^	1.605^***^	0.812^**^	0.806^**^
	(0.255)	(0.255)	(0.366)	(0.367)
Sex	0.187	0.194	−0.600	−0.589
	(0.287)	(0.288)	(0.508)	(0.511)
Party in government	0.349	0.343	−0.410	− 0.419
	(0.277)	(0.277)	(0.360)	(0.363)
Rol in house	−0.543	− 0.556	−14.642	−14.701
	(0.347)	(0.349)	(1,029.1)	(1,029.1)
Intercept	−1.614^***^	−1.636^***^	−0.515	−0.533
	(0.221)	(0.227)	(0.391)	(0.400)
Observations	450	450	182	182
Log Likelihood	− 204.023	− 203.924	− 106.220	−106.194
Akaike Inf. Crit.	420.045	421.848	224.439	226.388
Pseudo R2	0.38	0.38	0.2	0.2

Reference categories: EMP: Non-emigrant MP; EDMP: Non-emigrant MP; Period: for Ecuador, period 2009–2013 and, for Colombia, period 2010–2014; Sex: male; Party in government: party in opposition; Role in house: no role. Standard errors in parenthesis. ^**^p-value < 0.05;^***^p-value < 0.001. Source: Author’s Own

In the case of Ecuador, Model 1a shows that, as expected, being an EMP significantly increases the odds-ratio of dedicating floor speeches to approach an emigrant-related issue (odd-ratio = 4.30; *p*-value < 0.001). Interestingly, the model also shows that there is a difference between legislative periods: all other variables being equal, a speech given in the period 2013–2017 is more likely to be classified as emigrant-related than a speech pronounced during the period 2009–2013 (odd-ratio = 1.61; p-value < 0.001). Model 1b shows similar results as Model 1a. The results for Colombia are similar. Model 2a and 2b suggest that being an EMP is also correlated significantly with the probability of given an emigrant-related speech (odd-ratio = 2.69; p-value < 0.001). As in Ecuador, speeches given during the second legislative period (when Colombia added an extra emigrant representative) are more likely to be classified as emigrant-related (odd-ratio = 0.81 with a p-value < 0.05 in Model 2a; and odd-ratio = 0.81 with a p-value < 0.05 for Model 2b). Being an EDMP does not significantly increase the probability of giving an emigrant-related speech during the floor discussions in Colombia or Ecuador. Finally, beyond legislative period, control variables do not have any significant effect on the classification of a speech as an emigrant-related intervention.^12^

### The perception of EMPs

The quantitative analysis provides relevant insights into the work that EMPs conduct in their legislatures. However, there are still questions that cannot be answered through quantitative analysis. For this reason, I interviewed four EMPs of Ecuador and the two EMPs of Colombia.^13^ The goal of these interviews is twofold. Firstly, I use them to validate the information provided by the analysis of EMP interventions. Secondly, I use them to understand better the rationale that is behind the work that EMPs conduct in their legislatures.

The previous quantitative analysis shows that both Ecuadorian and Colombian EMPs address both emigrant-specific issues and other general issues during their floor interventions. This dual interest is confirmed by the interviews. When asked about their legislative priorities, Ecuadorian EMPs clearly responded that their main duty was to represent the interests of their constituency. They, for instance, identified as priorities the “Human Mobility Law”, the integration of returnees, or the improvement of consular services. However, all of the Ecuadorian interviewees also identified non emigrant-related issues such as international affairs, welfare policy, or women’s rights; as part of their legislative work. The interviews of Colombian EMPs also confirm this pattern. They addressed emigrant-related issues, such as the rights of the victims of the armed conflict living abroad, but were also notably involved in other topics. As one of the Colombian EMPs explained “... I am a Colombian parliamentarian. I am elected by the Colombian emigrants, but I am interested in the future of the country as a whole…”.^14^

Another important finding of the interviews is connected to the mainstreaming of emigrant-related issues in the legislature. First, all Colombian and Ecuadorian EMPs interviewed mentioned the importance of introducing a migrant perspective into regulations that were not explicitly connected to an emigrant agenda. In the words of a Colombian EMPs “…many laws bring some benefit for Colombians, but not for those who are abroad. Then comes the task, as in the National Development Plan to say, well, we present an amendment to include them”. Second, some of them also highlighted the importance of convincing other parliamentarians to bear the overseas population in mind when legislating: “…there are times when I have had to get into trouble with another representative member, but you either do it or the subject remains invisible” (Ecuadorian EMP).

Several EMPs from both countries mentioned that a big part of their work was monitoring the government and mediating on behalf the emigrant community to express their demands to the executive. As a Colombian EMP expresses it “… it is about awareness work, to create the awareness that there are Colombians abroad and they have to be involved (…) tell the Ministries, the institutions, that there are Colombians abroad and they have to work for them”.

Finally, the interviews confirmed that both Ecuadorian and Colombian EMPs campaigned abroad to get elected mostly on issues that affected the diaspora abroad, as captured by the dictionary codes (e.g. return policies, consulate functions, health care abroad; in the case of Colombia the reparation of the victims of the armed conflict abroad). Also, they confirmed that they conduct district work by keeping ties with their emigrant communities via social networks and overseas travels –the latter being mostly self-funded in Ecuador and only partially in Colombia.

## Conclusions

Research on the substantive representation of non-resident citizens in their states of origin is scarce. However, investigating this particular topic is essential to gain new insights about the formal-descriptive-substantive link of political representation. This study tested the existing theories about minority representation, such as critical mass, on a group –emigrants– that has, up to now, been neglected in the literature of political representation. I implemented an innovative mixed-methods research design that combines a quantitative text analysis of more than 35,000 floor speeches with personal interviews with emigrant special representatives. The detailed inspection of floor speeches emerged as a valid and valuable source of information to assess substantive representation. It allowed me to compare different groups of representatives without previous sampling, enhancing the robustness of the measurements.

Scholars have argued that a system of special representation for non-resident citizens is fair because it allows large communities of emigrants to participate in the political systems of their state of origin, while at the same time controlling the effect that emigrants could have over homeland politics (see Bauböck, 2007). My findings shed some light to this debate from an empirical perspective by comparing a system of ‘contained’ special representation and one of (almost) ‘proportional’ special representation.

Firstly, the findings show that the descriptive representation of emigrants leads to some degree of substantive representation (H1). Even though NEMPs target the emigrant community in their floor speeches, it was proved that EMPs dedicate relatively more of their time in plenary sessions tackling emigrant issues. Nevertheless, the degree of substantial representation differs considerably across both countries. In Colombia, where there are only one or two EMPs, the salience of emigration is significantly lower than in Ecuador, a country with six EMPs. In the same line, the statistical models also suggest that in both countries, being a member of the EMP group, increases the predicted probability of giving a speech explicitly related to emigrant policies. In line with H2a, this effect is stronger in Ecuador than in Colombia. The low salience in Colombia is evidence of the existence of a certain ‘containment effect’ (in line with H2a), but also can be influenced by other factors such as the prominence of other issues (e.g. the peace process). Furthermore, another possible explanation of this difference can be found in the formal rules that shape emigrant special representation in each country. The clear territorial connotation of the Ecuadorian system (divided into three external districts) increases the pressure on the special representatives to incorporate the demands of the non-resident community into their legislative work. This territorial link, however, is weaker in Colombia, where emigrant special representation was granted as a mechanism to give voice a minority group and not a territorial entity.

Secondly, the results do not show a clear marginalization effect of EMPs in any of the countries. In fact, the Colombian EMPs were shown to participate significantly more, on average, than other MPs. In Ecuador, the difference in the number of speeches given by EMPs and NEMPs does not follow a clear pattern.

Thirdly, the qualitative analysis suggests that, although EMPs prioritize emigrant issues in their legislative agenda, those are not the only substantive issues that they address. Thus, there is clear evidence that EMPs have a ‘mixed agenda’ that includes emigrant issues as a priority, but not exclusively so. Moreover, the analysis shows that they dedicate plenty of effort to introducing the migration perspective in non-emigrant specific topics (such as labor regulations, development plans or women rights), in monitoring the actions of the executive power regarding the wellbeing of emigrants and in mediating between the demands of their constituency and the government.

All in all, the results suggest that, as predicted, there is a certain ‘containment effect’ in the case of Colombia and that higher numbers of EMPs lead to a better substantive representation of the interests of non-resident citizens (H2a). There is no evidence to confirm that underrepresented numbers of EMPs represent better emigrant interests by holding an underlying symbolic power (H2b). However, these claims should be tested further by including more countries in the analysis and more legislative periods. Future research should also explore the effect of emigrant special representation on symbolic representation (Pitkin, 1967) or the overall effect of EMPs work on the legitimacy of the democratic system (a question posed already by other colleagues such as Bermudez, Lafleur, & Escriva, 2017).

Finally, on a methodological note, the study here presented had one limitation. For the Colombian case, the results are based on the work of two EMPs meaning that the findings can be highly affected by the profile of single individuals. Over time, when more legislative periods are concluded, this study should be replicated in order to prove if its findings stand the test of time.

## Appendix

### Treatment of floor speeches

In the case of Colombia, the protocols of the floor sessions were downloaded from the official website and, in the case of Ecuador, they were provided by the library of the National Assembly. The protocols were first transformed into plain text documents. Then, a recognition script based on python (using regular expressions) was rendered in order to mine the interventions of each parliamentarian. To prepare the speeches for the next step, namely dictionary coding, I followed the instructions proposed by Manning, Raghavan, and Schütze (2008) and Grimmer (2010).

Firstly, the method discards the order of the words in the speeches producing an output that contain unordered words. Although the order of the words is important to fully understand a text, the aim of this method is to identify the topic of the speeches and this “should be invariant of permutations of word order” (Grimmer, 2010, p. 6).

Subsequently, all words are converted to lowercase, and punctuation and stopwords (e.g. ‘however’, ‘because’ etc.) are removed. Contrary to the instructions given by Manning et al. (2008), I do not generate stems and I keep the original words. I also keep combination of words that are considered important for the analysis (e.g. “return policy” or “consular services”).

Computations are conducted in R, using the tm package.

## Footnotes

1 Depending on the legislative period, Colombian emigrants have been able to elect one or two representatives. For the period of analysis 2010–2014, emigrants had only one representative; and for the period 2014–2018, emigrants have two representatives.

2 6 members out of 137 (legislative period 2013–2017).

3 The number of seats in the Colombian House of Representatives has changed over time. It has passed from 1 to 2 and then again 1.

4 1 out of 166 members in the legislative period 2010–2014 and 2 out of 166 members in the legislative period 2014–2018.

5 This trend is also observable in the general turnout in Ecuador and Colombia. For instance, in the Colombian presidential elections of 2014, participation only reached 40.65%, while in the last Ecuadorian presidential elections of 2017, participated reached 81.63%.

6 In Ecuador, each representative can participate a maximum of two times in each debate (Asamblea Nacional, 2009 Art. 130). The rules of the Colombian House of Representatives limit to one per debate the interventions of individual representatives or two in the case of interventions given by speakers of constituted parliamentarians groups (El Congreso de Colombia, 1992 Art. 97).

7 From this sample, 94% of the Colombian speeches (period 2010–2014) and 96% (period 2014–2018) were classified correctly. For Ecuador, 96% of the speeches (period 2009–2013) and 98% (period 2013–2017) were classified correctly.

8 For the Ecuadorian case and for the period 2013–2017, the analysis only covers 307 floor sessions of the 345 that took place, which represent a total of 88.9% of the floor sessions. The protocols for the missing sessions were not publicly available.

9 Colombian districts designated as highly affected by emigration: Atlántico, Antioquía, Norte de Santander, Risaralda, Valle del Cauca and Cudinamarca. Ecuadorian provinces designated as highly affected by emigration: Azuay, Canar, El Oro, Guayas, Manabí and Pichincha.

10 It may be possible that the content of speeches is related to the party size. However, the preliminary analyses conducted show that, for the legislative periods analyzed, party size is highly correlated with the variable that captures if the MP is a member of the party in government or not. To avoid multicollinearity issues in the regression analysis, this variable has been not included in the final analysis.

11 Each model includes the data for the two legislative periods analyzed for each country.

12 In order to increase the internal validity of the regression results, I also implemented exact matching to compare EMPs with NEMPs who have the same score in control variables. The results of this exact matching show similar results as the models presented in Table 6.

13 I interview EMPs from Colombia (legislative periods 2010–2014 and 2014–2018) and Ecuador (legislative periods 2009–2013 and 2013–2017).

14 Excerpts are translated from the original in Spanish by the author.
